# Behavioral and multiomics analysis of 3D clinostat simulated microgravity effect in mice focusing on the central nervous system

**DOI:** 10.1038/s41598-025-90212-y

**Published:** 2025-02-17

**Authors:** Li Zhou, Chenchen Song, Hu Yang, Lianlian Zhao, Xianglei Li, Xiuping Sun, Kai Gao, Jianguo Guo

**Affiliations:** Key Laboratory of Human Disease Comparative Medicine, Chinese Ministry of Health, Key Laboratory for Animal Models of Emerging and Reemerging Infectious Diseases, Institute of Laboratory Animal Science, Chinese Academy of Medical Sciences and Comparative Medicine Center, Peking Union Medical College, Beijing, China

**Keywords:** Microgravity, Three-dimensional clinostat, Brain, Transcriptomics, Microbiomics, Serum metabolomics, Animal model, Neuroscience, Zoology, Risk factors, Environmental impact, Transcriptomics, Physiology

## Abstract

A study was conducted to evaluate the three-dimensional clinostat simulated microgravity effect on mouse models, focusing on the central nervous system. Eighteen mice were divided into three groups: control, survival box, and clinostat + survival box. Behavioral tests, femur micro-CT, brain transcriptomics, serum metabolomics, and fecal microbiomics were performed. Results showed decreased activity, altered gait, enhanced fear memory, bone loss, immune/endocrine changes in brain transcriptome, and altered metabolic pathways in serum and gut microbiota in clinostat-treated mice. The model closely mimics spaceflight-induced transcriptome changes, suggesting its value in studying microgravity-related neurological alterations and highlighting the need for attention to emotional changes in space.

## Introduction

Space exploration is a long-term dream and pursuit of humanity, and it is also one of the important fields of technological development. In long-term space flight, astronauts face many problems. One of them is the reduction of gravitational force. Terrestrial life has evolved under the influence of gravity for millions of years, and long-term microgravity represents unusual stress experiences that can cause physiological and morphological changes^1^. Numerous studies have shown that long-term weightlessness can lead to various physiological and pathological changes, such as the skeletal and muscular system, cardiovascular system, immune system, and nervous system^2^. Prolonged exposure to microgravity during extended space missions poses various physiological challenges to astronauts, necessitating thorough research into the effects of microgravity on the human body. One of the crucial issues is the impact of long-term spaceflight on brain function. Studies have shown that astronauts experience changes such as ataxia, posture disorders, perceptual illusions, fatigue, and cognitive impairment^3^. The high cost and technical issues related to spaceflight have stimulated the development of ground models that reasonably simulate the effects of space flight. Currently, tail-suspension hindlimb unloading is the most classic and widely used model, particularly those affecting the musculoskeletal system^4^. However, this model has certain limitations, that is, it can only be applied locally, and cannot be applied to the whole experimental animal. Besides, there are physiological differences between hindlimb unloading models and spaceflight, especially in terms of somatosensory input, spatial orientation, and vestibular disruption^5^.

Three-dimensional (3D) clinostat is a device that generates multi-directional G forces. By controlling the rotation on both axes, it can offset the cumulative gravity vector at the center of the device^6^. Previous studies have shown that 3D clinostat have been widely used on plants, cells and Caenorhabditis elegans to simulate microgravity effects^7–9^. Our previous studies suggest that the central nervous system probably changed in mice trained with 3D clinostat^10^. Therefore, the aim of our work is to systematically assess 3D clinostat simulated microgravity effect in mice by behavioral and multi group analysis, focusing on the changes in central nervous system and cognitive function.

## Methods

### Mice and treatment

**Fig. 1:**
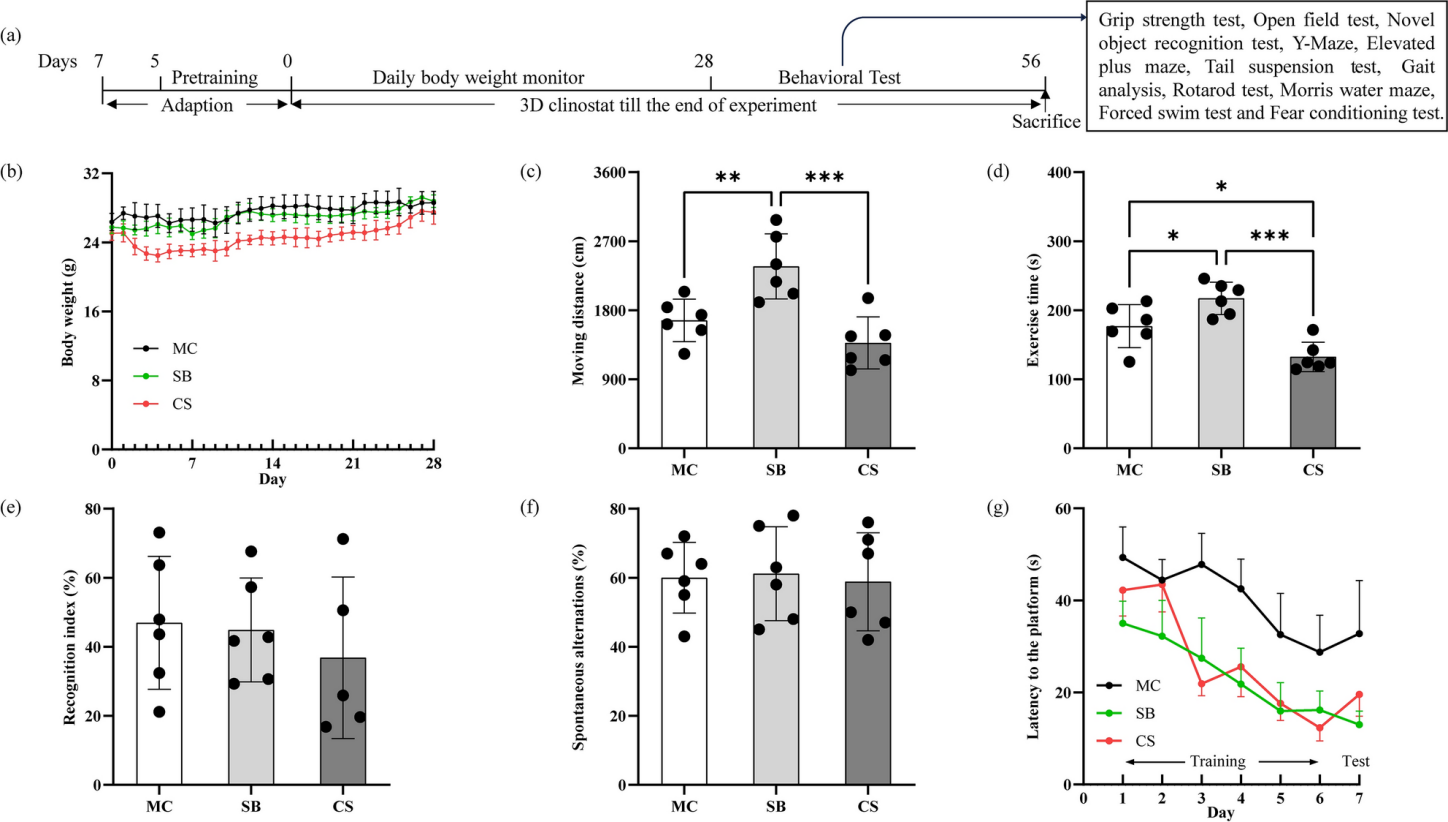
(**a**) Flowchart of the experimental procedures and the behavioral experiments are conducted in the order of arrangement; (**b**) Changes in mice body weight; (**c**) Total moving distance of mice in open field test; (**d**) Exercise time of mice in open field test; (**e**) Recognition index of mice in novel object recognition test; (**f**) Spontaneous alternations index in Y maze; (**g**) Latency to the platform time in Morris water maze. MC: ordinary cage control group; SB: survival box control group; CS: 3D clinostat model group. The data are presented as the mean ± SD, *P < 0.05, **P < 0.01, ***P < 0.001, n = 6 for each group.

10-week-old male C57BL/6J mice were purchased from Beijing HFK Bioscience Co., Ltd and kept in the experimental room at temperature 22 ± 2 °C, humidity 40 ± 5%, under a 12-h light/dark cycle. The mice were randomly divided into three groups: (1) MC group with mice housed in ordinary cage (MC, n = 6); (2) SB group with mice housed in survival box (SB, n = 6); (3) CS group with mice housed in survival box receiving 3D clinostat treatment (CS, n = 6). The structure of the 3D clinostat and the survival box (shown in Supplementary Figure S1) has been described in detail in our previous study^10^. The rotation modes are as follows: random rotation speed, 0–10 rpm; speed resolution, 0.1 rpm. Before the study, all mice were acclimated to the facility environment for 2 days and then adaptive training was carried out for 5 days to alleviate stress and adapt to the jelly like diet containing 59% water, 1% agar, and 40% purified feed. During training, the CS group underwent adaptive rotation for 1 h, 2 h, 4 h, 8 h, and 12 h each day, while the MC group was always placed in ordinary cage and the SB group was always placed in survival boxes without any treatment. The formal experiments began at 11 weeks old. The CS group experienced continuous simulated microgravity effect on clinostat for 8 weeks with the exception of cleaning cages and changing food for approximately 20 min per day; the other two groups of mice always lived in corresponding living facilities in the same room. To avoid the interference of acute stress, weight monitoring showed that the body weight of mice in the CS group gradually returned to the control group level after 28 days, suggesting that the mice had fully adapted to the 3D clinostat. Starting from the fifth week, mice underwent a series of behavioral experiments. Following each behavioral experiment, the mice were promptly placed into a 3D clinostat to ensure continuous microgravity effect stimulation. After 8 weeks, the mice were euthanized dissected for sampling at the end of the experiment (Fig. 1a). Animal care was provided in accordance with institutional guidelines and all animal studies were performed in compliance with the ARRIVE guidelines and were approved by the Ethics Committee of the Laboratory of Animal Science of Peking Union Medical College (IACUC-20220415).

### Mouse behavioral tests

The sequence of the behavioral experiments was arranged as follows: Grip strength experiment (day 29), Open field test (day 30), Novel object recognition test (day 30–32), Y-Maze (day 33), Elevated plus maze (day 34), Tail suspension test (day 35), Gait analysis (day 36–40), Rotarod test (day 42–44), Morris water maze (day 45–51), Forced swim test (day 52) and Fear conditioning test (day 53–56). Each mouse was tested individually and then put back into the 3D clinostat as soon as possible after the test to minimize the time spent disengaging from the rotation.

Detailed methods of behavioral testing are as follows:

#### Grip strength experiment

The evaluation of grip strength was conducted using a grip strength meter. Each animal was individually placed on the grip strength board and pulled by the tail, prompting them to grasp the bar. The rodents instinctively grasp anything in order to prevent this involuntary backward movement until the pulling force exceeds their grip strength. The maximum value of grip strength was recorded using the Bioseb system. Each animal was tested three times repeatedly, and the mean value was calculated. During the behavioral testing time, each mouse was removed from the 3D clinostat device for about 15 min for testing, and then quickly returned to the 3D clinostat device after testing.

#### Open field test

The open field was an empty square environment, with a size of 50 cm × 50 cm × 30 cm. A central area was marked in the open field, centered in the middle of the empty space, with a square length of 16 cm zone. The fringe area was 10 cm wide. The animals were placed in the middle of the central area and observed for five minutes. Behavioral parameters including velocity, distance traveled, and time spent in the central and fringe areas were recorded by a computerized video tracking system (Ethovision XT; Noldus, Information Technology, The Netherlands). The open field apparatus was cleaned after each session using 70% ethanol and allowed to dry between tests. During the behavioral testing time, each mouse was removed from the 3D clinostat device for about 20 min for testing, and then quickly returned to the 3D clinostat device after testing.

#### Novel object recognition test

The novel object recognition test was used to assess short- and long-term recognition memory. The experimental procedure consisted of three stages. On the first day, there was an adaptation period without any toy bricks. On the second day, familiarization took place with two toy bricks (2 cm × 2 cm) in opposite corners, both with the same shape, size, and color. On the third day, the test period occurred, with one of the toy bricks changed to a different shape and color. The camera on top of the box was used to record how long it took for a mouse to examine the new object (TN) and the familiar object (TF), allowing a total of five minutes for exploring the two distinct objects. The recognition index was calculated as follows: Recognition index = (TN/(TN + TF)) × 100%. A higher recognition index reflected better short- and long-term recognition memory abilities in animals within this group. The behavioral test time was about 20 min per day for each mouse to be removed from the 3D clinostat for testing, and then quickly returned to the 3D clinostat after testing.

#### Y-maze

In the Y-maze test, each mouse was placed at the end of one arm of the 'Y' and allowed to explore the maze freely for five minutes. The sequence of entry of the mice in the arms was recorded to calculate the percentage of change. The mouse entered three different arms in turn, which was counted as one alternation. Spontaneous alternations (%) = Number of spontaneous alternations/(Total number of arm entries − 2) *100%. Higher spontaneous alternations indicated better learning and memory ability. During the behavioral testing time, each mouse was removed from the 3D clinostat device for about 20 min for testing, and then quickly returned to the 3D clinostat device after testing.

#### Elevated plus maze

During the Elevated Plus Maze (EPM) test, the maze was composed of four arms (30 cm × 5 cm). Two opposite arms were closed, enclosed by lateral walls (depth 5 cm), and the remaining two arms were open and lacked walls. The four arms were connected by a central area (5 cm × 5 cm), and the maze was elevated 50 cm from the floor. During the test, mice were placed on the central square platform and allowed to explore the maze for 5 min. The following parameters were recorded and analyzed: the number of entries into open/closed arms, the time spent in the open/closed arms, and the total number of arm entries. These data were recorded by a camera and analyzed using a computerized video tracking system.

The anxiety index was calculated as the ratio of open arm time and entries to total time and total entries. The open arm entries percent was calculated as entries into the open arm divided by the total number of arm entries (entries into the open arm + entries into the closed arm). During the behavioral testing time, each mouse was removed from the 3D clinostat device for about 20 min for testing, and then quickly returned to the 3D clinostat device after testing.

#### Tail suspension test

The Tail Suspension Test was performed as previously described^11^. Adhesive tape was applied to the tail, approximately 1 cm from the tip, and the mouse was suspended for 6 min. After a 2 min habituation period, the immobility time was recorded during the final 4 min using Tail Suspension Real-Time Analysis (System-2.0). During the behavioral testing time, each mouse was removed from the 3D clinostat device for about 20 min for testing, and then quickly returned to the 3D clinostat device after testing.

#### Gait analysis

The gait of naturally moving mice was analyzed using CatWalk XT (Noldus Information Technology, Netherlands), as previously described^12^. This gait analysis system consists of a glass walkway floor illuminated with a green light that is completely internally reflected in the glass, a standard charge-coupled device (CCD) camera underneath, and software for recording and analyzing the data obtained from mouse paws. A darkroom box was used at the end of the walkway to create an incentive for the mice to cross the field, where the entrance to their home cage is placed. The mice were placed in the front of the start zone of the walkway and were trained to cross the walkway with five accomplished runs per day on five consecutive days. The test was started on the fifth day, and each mouse was tested three times. The successful run was defined as a mouse walking across the runway without any hesitation. The walkway was cleaned thoroughly with 70% ethanol between each animal. Gait parameters of footprints were automatically generated (left front, LF; left hind, LH; right front, RF; and right hind, RH). The behavioral test time was about 20 min per day for each mouse to be removed from the 3D clinostat for testing, and then quickly returned to the 3D clinostat after testing.

#### Rotarod test

The rotarod apparatus (Model: 47600, Italy) was used to assess the locomotor abilities of rodents, particularly their coordination and endurance. Mice were subjected to a training session of four trials per day for 3 days, the rotarod gradually accelerated from 5 to 40 rpm within 120 s until the mouse fell off. Starting from the 4th day after training, formal testing experiments were conducted. During test trial, the rotarod gradually accelerated from 5 to 40 rpm within 300 s. The latency at which the mouse fell from the rod (or a maximum of 5 min) was recorded. The average latency from three trials was determined. During the behavioral testing time, each mouse was removed from the 3D clinostat device for about 30 min for testing, and then quickly returned to the 3D clinostat device after testing.

#### Morris water maze

The water maze was divided into four quadrants and contained a platform that remained submerged under 1–1.5 cm of water. During the training trials, mice were placed in the water from the opposite quadrant of the platform. Successful navigation to the platform was considered when the mouse found it within 1 min. If the mouse failed to find the platform within this time limit, a guidance rod was used to guide the mouse to stay on the platform for 10 s and the latency was recorded as 60 s. Training trials were conducted for three trial sessions each day for six consecutive days. During probe trials, the platform was removed from the maze. Frequency of visits to the platform, time spent in the platform quadrant, cumulative duration in the target zone, and latency to the platform were used as indicators of memory performance. The behavioral test time was about 30 min per day for each mouse to be removed from the 3D clinostat for testing, and then quickly returned to the 3D clinostat after testing.

#### Forced swim test

The forced swim test was performed as previous studies^13^. Each mouse was individually placed into a non-transparent cylindrical plastic container (height of 25 cm; diameter of 18 cm) containing 15 cm of water at 23–25 °C. The test duration was 6 min, and immobility was analyzed during the last 4 min. Immobility behavior was considered by inactive time when the animal remained floating passively without any movements, with the exception of minimal movements to keep the head above water. During the behavioral testing time, each mouse was removed from the 3D clinostat device for about 20 min for testing, and then quickly returned to the 3D clinostat device after testing.

#### Fear conditioning test

Both contextual and cued fear conditioning protocols were implemented as previous studies^14^. On the first day, mice were introduced to a Plexiglas training cage under constant illumination for 5 min. On the second day, animals were allowed to adapt for 180 s in the box before commencing three to five rounds of circulation training. Auditory stimulation (30 s, 5 kHz, 70 dB) was then administered, followed by the delivery of electric current stimulation (0.65 mA, 1 s). The intervals between each training session were 30–60 s. Animals were allowed to remain in the cage for 30 s before being returned to their home cage. Twenty-four hours later, a contextual fear conditioning test was conducted by placing animals back into the same training cage without any sound or electric current stimulation, and their freezing behavior was recorded over a 330 s period. On the fourth day, cued fear conditioning began. The background and smell of the boxes were changed, and after 180 s of adaptation in the box, only auditory stimulation (5 kHz, 70 dB) was presented. Freezing behavior was then recorded over a 420 s period. Prior to each animal’s placement, the fear-conditioning box was thoroughly cleaned with 70% ethanol. The behavioral test time was about 20 min per day for each mouse to be removed from the 3D clinostat for testing, and then quickly returned to the 3D clinostat after testing.

### Brain transcriptomics

Brain samples were bisected along the plane separating the hemispheres. For each brain, one hemisphere was selected for the generation of mRNA sequencing data, following a methodology similar to that described in the previously published paper^15^. Total RNA extraction was carried out using the TRIzol reagent (Invitrogen, CA, USA), following the manufacturer’s protocol. To assess the quality and quantity of the extracted RNA, the NanoDrop 2000 spectrophotometer (Thermo Scientific, USA) was employed. The integrity of the RNA was then evaluated using the Agilent 2100 Bioanalyzer (Agilent Technologies, Santa Clara, CA, USA). Subsequently, RNA libraries were constructed with the VAHTS Universal V6 RNA-seq Library Prep Kit, strictly adhering to the manufacturer’s instructions.

The transcriptome sequencing and subsequent analysis were conducted by OE Biotech Co., Ltd. (Shanghai, China). The libraries were sequenced on an Illumina Novaseq 6000 platform, generating 150 bp paired-end reads. The raw reads in fastq format underwent an initial processing step using fastp. During this process, low-quality reads were filtered out to obtain clean reads suitable for further analysis. The clean reads were then mapped to the reference genome using HISAT2. To quantify gene expression levels, the FPKM of each gene was calculated and the read counts for each gene were obtained via HTSeq-count. To evaluate the biological duplication of the samples, Principal Component Analysis (PCA) was performed using the R programming language (version 3.2.0). For the differential expression analysis, the DESeq2 package was utilized. Genes were considered significantly differentially expressed if they met the criteria of a *P* value < 0.05 and a fold change > 1.5 or fold change < 0.667. Bioinformatic analysis and graphics was performed using the OECloud tools at https://cloud.oebiotech.com/task/.

The hindlimb unloading mice and real spaceflight microgravity mice brain transcriptome data have been sourced from published data. Specifically, the brain RNA-seq data of hindlimb unloading mice, which underwent hindlimb unloading for 2 weeks and had their total RNA extracted from each brain using Trizol for transcriptome analysis (GeneLab ID: GLDS-32, 10.26030/jpyz-fn46), and the data from spaceflight mice, that were housed in the Rodent Habitat for 39–42 days, euthanized in space (mice were anesthetized by ketamine/xylazine/acepromazine and subjected to cardiac puncture followed by cervical dislocation), and had their whole carcasses stored at − 80 °C during the Rodent Research-3 mission (GeneLab ID: GLDS-352, 10.26030/jm59-zy54), are both accessible within the NASA Open Science Data Repository (https://osdr.nasa.gov/bio). Differential expression analysis was performed using the DESeq2. *P* value < 0.05 and foldchange > 1.5 or foldchange < 0.667 was set as the threshold for significantly differential expression gene. Bioinformatic analysis and graphics was performed using the OECloud tools at https://cloud.oebiotech.com/task/.

To compare the differences between three models, namely the 3D clinostat model (CS), hindlimb unloading model (HU), and space flight mice (FL), we conducted a distance comparison of transcriptome changes. Select shared genes from transcriptome data for analysis, and obtain log_2_FoldChang values for each gene, namely FC_CS_(1,2,3…n), FC_HU_(1,2,3…n), FC_FL_(1,2,3…n), and then calculate the brain transcriptome distance between each model, S_CS/FL_ = $\sum_{1}^{n}|{FC}_{cs}n-{FC}_{FL}n|$, S_HU/FL_ = $\sum_{1}^{n}|{FC}_{HU}n-{FC}_{FL}n|$, S_CS/HU_ = $\sum_{1}^{n}|{FC}_{cs}n-{FC}_{HU}n|$. The similarity of the three models can be compared by the magnitude of the distance value S. By utilizing the multiple expression changes of each gene in different models relative to their respective control groups, a heatmap can be drawn to visually display and compare the differences in the brain transcriptome of each model.

### Serum metabolomics

To extract metabolites, 400 μl of cold extraction solvent methanol/acetonitrile/H_2_O (2:2:1) was added to 100 μl of serum sample and adequately vortexed. The samples were then incubated on ice for 20 min and centrifuged at 14,000*g* for 20 min at 4 °C. The supernatant was dried in a vacuum centrifuge. For LC–MS analysis, the samples were re-dissolved in 100 μL acetonitrile/water (1:1, v/v) solvent and centrifuged at 14,000*g* at 4 °C for 15 min, then the supernatant was injected. For the untargeted metabolomics of polar metabolites, extracts were analyzed using a quadrupole time-of-flight mass spectrometer (Sciex Triple TOF 6600) coupled to hydrophilic interaction chromatography via electrospray ionization by the Shanghai Applied Protein Technology Co. Ltd. Preparation of QC samples by mixing aliquots of all samples. The raw LC–MS data obtained were processed using Proqenesis QI Software (Waters Corporation Milford, USA). For statistical analysis, we made use of both univariate (Student’s t-test and fold change analysis) and multivariate (principal component analysis and orthogonal partial least squares discriminant analysis) method. The variable importance in projection (VIP) scores > 1 and *P* values < 0.05, and fold change > 2 or < 0.5 were used to filter the differential metabolites. For the KEGG annotation of metabolic pathways, the metabolites were compared against the online KEGG database using BLAST to retrieve their COs, and then mapped to the corresponding KEGG pathways^16–18^.

### Analysis of the diversity of gut microbiota in feces

OE biotech Co., Ltd. (Shanghai, China) conducted the 16S amplicon sequencing and analysis. Briefly, genomic DNA was extracted from fecal samples using the DNeasy PowerSoil Kit (QIAGEN, Germany) and its concentration and purity were assessed by NanoDrop 2000 (Agilent, USA). The extracted DNA was used as template for PCR amplification of bacterial 16S rRNA with universal primers for V3-V4 variable regions. Sequencing was performed by Illuminaa NovaSeq 6000 with 250 bp paired-end reads. The raw sequencing data, in FASTQ format, were preprocessed using Cutadapt software to remove adapters. Following trimming, low-quality sequences were filtered out, and the remaining reads were denoised, merged, and chimera-checked using DADA2 with default QIIME2 parameters. The software ultimately output representative reads and an ASV abundance table. The representative read of each ASV was selected using the QIIME2 package, and all representative reads were annotated against the Silva database using q2-feature-classifier.

### Micro-CT imaging of bone

The femoral bone was dissected of soft tissues, fixed in formalin and the distal metaphysis scanned with a Siemens INVEON scanner using the following settings: tube voltage, 60 kV; tube current, 400 µA; and exposure time, 800 ms over 360° rotation. The Feldkamp filtered back-projection algorithm was used to reconstruct the images to generate 3D reconstructions. Images acquired from the scanner were viewed and calculated morphometric parameters with INVEON Workplace software.

### Statistical analysis

The data are shown as means ± SD. GraphPad Prism 10.0 software was used for statistical analysis. Two-way ANOVA followed by post-hoc test evaluated the significance of differences between groups. A difference was considered significant if the *P* value < 0.05.

## Results

### Effects of 3D clinostat on the body weight and general status in mice

Figure 1b showed the daily changes in weight of mice over the first 4 weeks. The SB group mice exhibited a weight profile similar to that of the MC group, indicating good adaptability to survival box. In contrast, the CS group mice undergoing 3D clinostat initially experienced a significant weight loss during the first 4 days. However, these mice gradually adapted to the 3D clinostat and their weight stabilized, followed by a slow recovery to levels similar to those of the SB control group and MC control group. Overall, the mice appeared healthy throughout the study period, indicating good adaptability to the 3D clinostat instrument.

### The impact of 3D clinostat on cognitive and motor related behavior

**Fig. 2:**
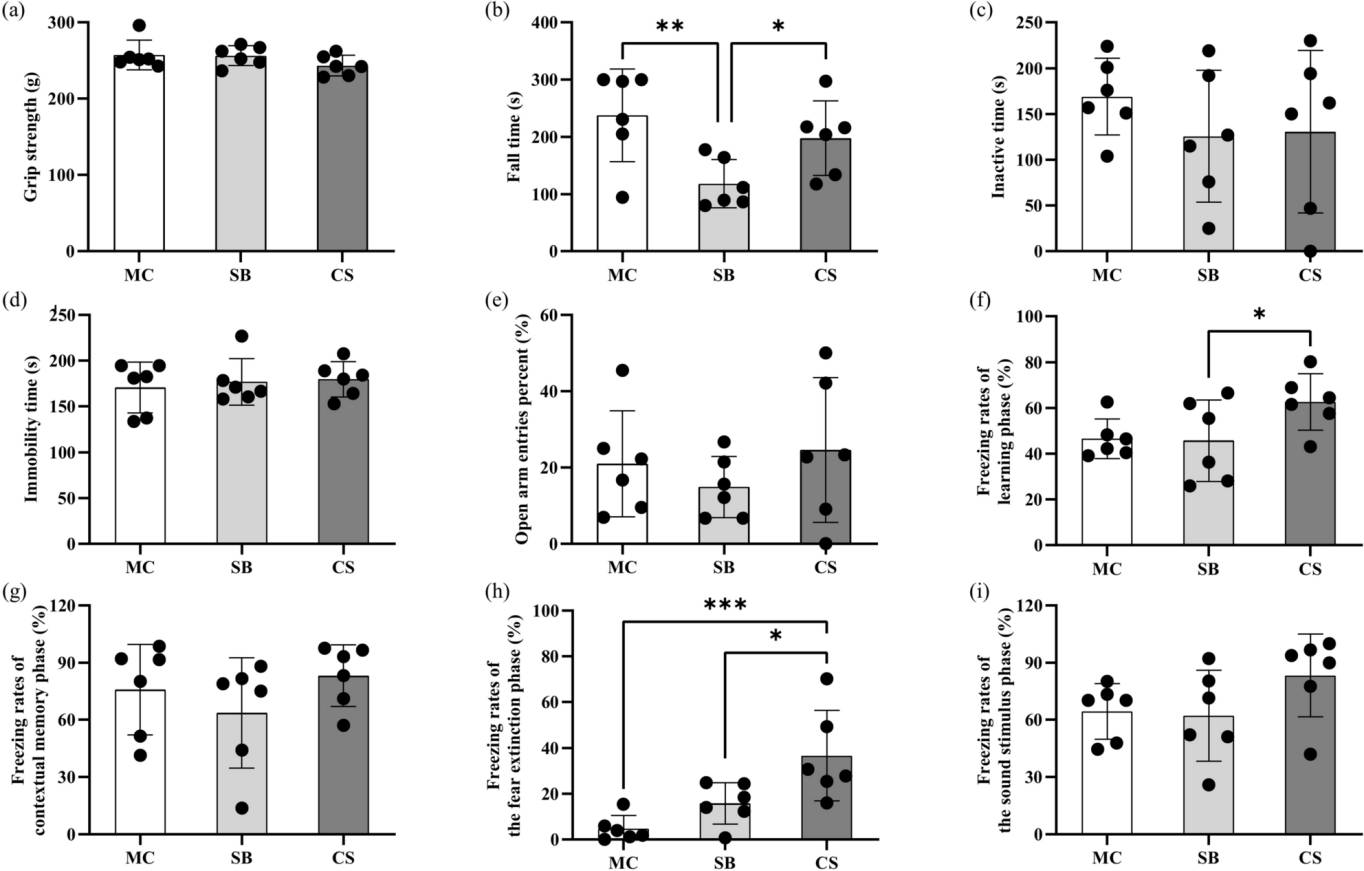
Behavioral test results. (**a**) Grip strength of limbs in the grip strength test; (**b**) Fall time in the rotarod test; (**c**) Inactive time in the forced swim test; (**d**) Immobility time in the tail suspension test; (**e**) Percentage of open arm entries in the elevated plus maze; (**f**–**i**) Freezing rates of mice in the fear conditioning test during the learning phase (**f**), contextual memory phase (**g**), fear extinction phase (**h**), and the conditioned sound stimulus phase (i). MC: ordinary cage control group; SB: survival box control group; CS: 3D clinostat model group. The data are presented as the mean ± SD, *P < 0.05, ** P < 0.01, *** P < 0.001, n = 6 for each group.

We conducted a series of behavioral experiments to observe the 3D clinostat simulated microgravity effect on cognitive and motor related behaviors in mice. The results showed 3D clinostat treatment significantly reduced mice activity and exploratory desire, indicated by the significant reduction in movement distance and exercise time in the open field test as shown in Fig. 1c–d, as well as in other behavioral experiments, including Y Maze, elevated plus maze and novel object recognition test (data not shown). In terms of learning and memory, there were no statistically significant differences in the recognition index in novel object recognition test (Fig. 1e), the spontaneous alternations index in the Y maze (Fig. 1f), and the latency to the platform in the Morris water maze (Fig. 1g) between CS and SB group, suggesting that 3D clinostat did not have a significant impact on the learning and memory. Regarding motor ability, a series of tests including grip strength test, rotarod test, and gait analysis were conducted. The results of the grip strength test revealed no notable differences among the three groups of mice (Fig. 2a). In the rotarod test, mice in the SB group exhibited a significantly shorter latency to fall compared to those in the MC group. This shorter latency may be attributed to the restricted activity space within the survival box for the SB group mice. Conversely, the CS group mice displayed a significantly longer latency to fall compared to the SB group, potentially due to their gradually acquired balancing ability through the 3D clinostat process (Fig. 2b). Besides, gait analysis revealed significant changes in certain gait parameters in the CS group compared to the SB group, indicating that prolonged exposure to the 3D clinostat had a notable impact on the vestibular balance system (Table 1, with the raw data shown in Supplementary Table S1). In terms of anxiety and depression, we conducted a series of experiments including the forced swim test, tail suspension test, and elevated plus maze. The results indicated that there were no statistically significant differences among the three groups of mice in terms of the duration of immobility in both the forced swim test and tail suspension test, as well as the percentage of open arm entries in the elevated plus maze. These findings suggest that exposure to the 3D clinostat did not result in significant depression or anxiety tendencies (Fig. 2, c-e). However, in the fear conditioning test, the 3D clinostat seemed to induce a trend towards increased sensitivity to fear behavior and enhanced fear memory. During the fear memory learning phase, the CS group mice exhibited a notable increase in freezing rates (Fig. 2f), coupled with a tendency of increased freezing rates in the contextual memory phase (Fig. 2g). Especially during the extinction phase, CS mice still demonstrated a significant increase in freezing rate, indicating enhanced fear memory and weakened extinction (Fig. 2h). Additionally, an increasing trend in freezing rate was also observed in the CS group mice during the subsequent sound stimulus phase (Fig. 2i). Upon comprehensive analysis of the behavioral research, it can be concluded that the 3D clinostat did not exert a notable influence on learning and memory processes. However, it potentially influenced the fear behavioral, along with subtle alterations related to motor balance.

**Table 1:** Gait Parameters with differences between the CS and SB groups.

Gait parameters	MC (Mean ± SD)	SB (Mean ± SD)	CS (Mean ± SD)	*P* (CS vs SB)	*P* (CS vs MC)	*P* (SB vs MC)
RF_PrintArea_(cm^2^)_Mean	0.272 ± 0.05	0.33 ± 0.051	0.248 ± 0.04	0.011	0.379	0.075
RH_MaxContactMaxIntensity_Mean	188.768 ± 6.89	183.125 ± 11.22	161.284 ± 15.857	0.02	0.003	0.319
RH_PrintLength_(cm)_Mean	0.839 ± 0.067	0.9 ± 0.069	0.816 ± 0.056	0.045	0.544	0.151
RH_PrintWidth_(cm)_Mean	0.656 ± 0.065	0.69 ± 0.044	0.62 ± 0.032	0.01	0.24	0.315
RH_MaxIntensity_Mean	193.748 ± 5.502	190.474 ± 7.926	167.327 ± 14.86	0.007	0.002	0.425
RH_MeanIntensityOfThe15MostIntensePixels_Mean	140.141 ± 8.092	133.75 ± 12.955	117.26 ± 12.025	0.045	0.003	0.33
LF_MaxContactArea_(cm^2^)_Mean	0.27 ± 0.03	0.282 ± 0.03	0.203 ± 0.038	0.003	0.007	0.502
LF_MaxContactMaxIntensity_Mean	165.715 ± 7.093	163.407 ± 7.699	141.793 ± 12.683	0.005	0.002	0.601
LF_MaxContactMeanIntensity_Mean	73.476 ± 3.211	74.367 ± 1.985	68.693 ± 3.993	0.011	0.045	0.576
LF_PrintLength_(cm)_Mean	0.888 ± 0.041	0.899 ± 0.078	0.762 ± 0.086	0.016	0.009	0.756
LF_PrintWidth_(cm)_Mean	0.754 ± 0.024	0.724 ± 0.063	0.644 ± 0.049	0.032	0.001	0.311
LF_PrintArea_(cm^2^)_Mean	0.329 ± 0.023	0.343 ± 0.036	0.241 ± 0.043	0.001	0.001	0.464
LF_MaxIntensity_Mean	174.92 ± 5.798	172.154 ± 8.777	150.079 ± 12.159	0.005	0.001	0.534
LF_MeanIntensity_Mean	76.797 ± 3.606	77.881 ± 2.337	72.247 ± 3.764	0.011	0.058	0.55
LH_MaxContactAt_(%)_Mean	29.644 ± 5.177	33.255 ± 5.425	44.056 ± 4.805	0.004	0.001	0.265
LH_MaxContactMaxIntensity_Mean	183.908 ± 10.444	183.98 ± 12.199	162.862 ± 10.968	0.01	0.007	0.991
LH_MaxIntensity_Mean	190.195 ± 7.764	191.693 ± 10.184	168.907 ± 11.337	0.004	0.004	0.78
LH_MeanIntensity_Mean	86 ± 3.002	85.876 ± 3.614	80.019 ± 1.755	0.005	0.002	0.95
LH_MeanIntensityOfThe15MostIntensePixels_Mean	135.542 ± 11.122	134.115 ± 9.127	116.662 ± 6.608	0.004	0.005	0.813
LH_StrideLength_(cm)_Mean	9.191 ± 2.131	10.083 ± 2.434	7.417 ± 0.962	0.032	0.093	0.514
BOS_HindPaws_Mean_(cm)	2.474 ± 0.108	2.477 ± 0.124	2.121 ± 0.142	0.001	0.001	0.966
PhaseDispersions_LH- > RH_Mean	48.759 ± 4.405	41.419 ± 7.088	48.738 ± 2.503	0.038	0.992	0.057
Couplings_LH- > LF_Mean	51.895 ± 2.686	57.778 ± 7.854	49.51 ± 3.672	0.042	0.228	0.113

LF- the left forelimb; RF- the right forelimb; LH- the left hindlimb; RH- the right hindlimb; MC: ordinary cage control group; SB: survival box control group; CS: 3D clinostat model group. n = 6 for each group.

### The impact of 3D clinostat on femur parameter

**Fig. 3:**
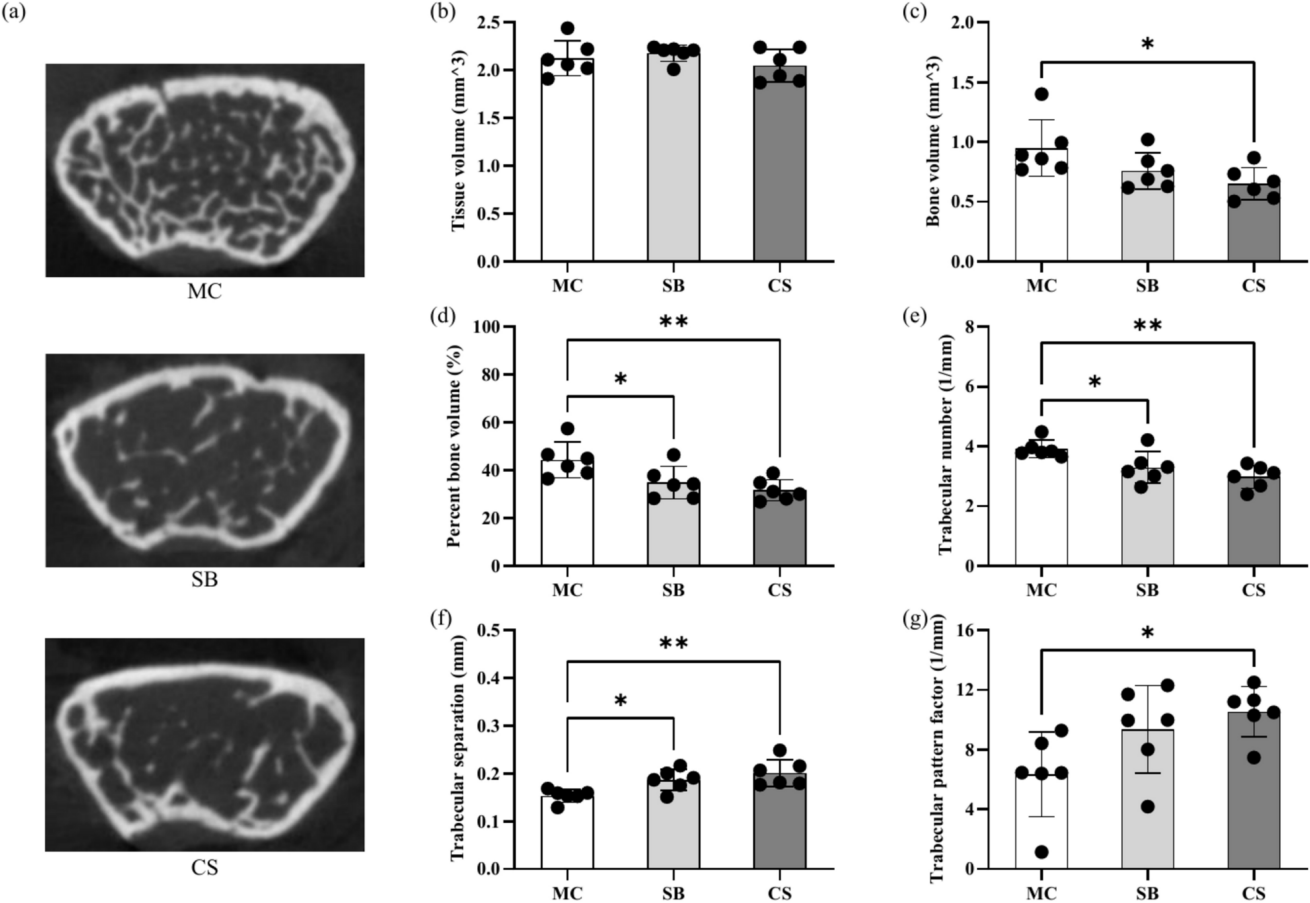
Changes in femoral bone parameters by micro-CT scanning. (**a**) Typical micro-CT cross-sectional images of femoral bone; (**b**–**g**) Changes in bone parameters including Tissue volume (**b**), Bone volume (**c**), Percent bone volume (**d**), Trabecular number (**e**), Trabecular separation (**f**), and Trabecular pattern factor (**g**) measured after 3D reconstruction. MC: ordinary cage control group; SB: survival box control group; CS: 3D clinostat model group. The data are presented as the mean ± SD, *P < 0.05, ** P < 0.01, n = 6 for each group.

To investigate the effects of 3D clinostat on bone metabolism, we utilized micro-CT to scan the femurs of mice and analyzed changes in parameters such as bone volume, tissue volume, trabecular pattern factor, trabecular number, and trabecular separation. The results revealed that both SB and CS group of mice exhibited significant bone loss compared to the MC group, with a notable decrease in bone volume and trabecular number, as well as a significant increase in trabecular pattern factor and trabecular separation. Furthermore, compared to the SB group, the CS group displayed a trend of further aggravating bone loss (Fig. 3).

### The impact of 3D clinostat on brain transcriptome

**Fig. 4:**
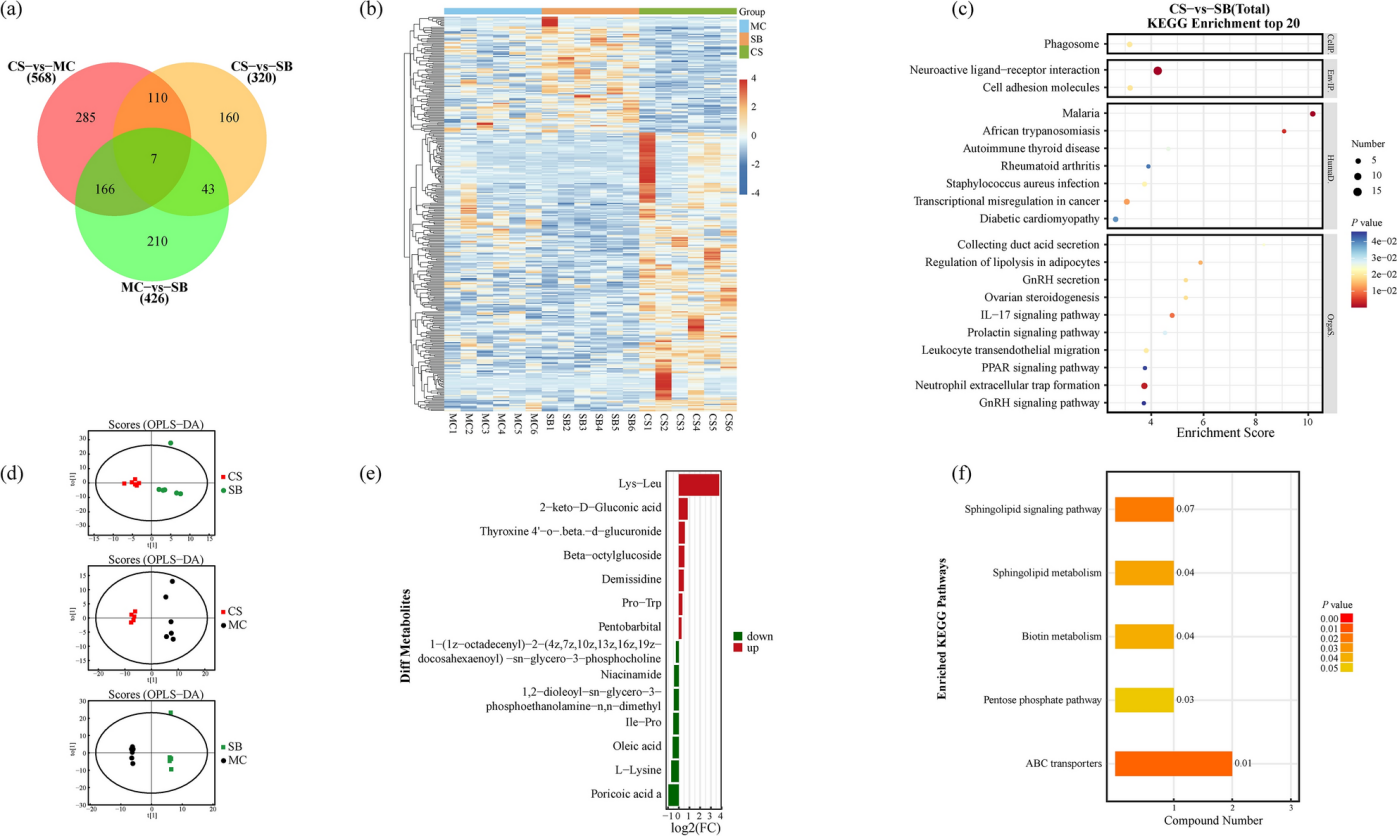
Alterations in brain transcriptome and serum metabolome of mice subjected to 3D clinostat. (**a**) Venn diagram illustrating differentially expressed genes between pairwise comparisons; (**b**) Heatmap of differentially expressed genes exhibiting significant differences between the CS and SB groups; (**c**) Main KEGG pathways affected between the CS and SB groups; (**d**) OPLS-DA analysis of serum metabolites between groups; (**e**) Upregulated and downregulated differential serum metabolites between the CS and SB groups; (**f**) KEGG analysis of serum metabolites revealing the main pathways affected between the CS and SB groups. MC: ordinary cage control group; SB: survival box control group; CS: 3D clinostat model group.

To evaluate the impact of 3D clinostat on the central nervous system, we conducted whole brain transcriptome sequencing. The results showed that there were 568, 320, and 426 significantly different genes between each group respectively, as shown in Fig. 4a. Figure 4b displays a heatmap of gene expression among the three groups for 320 genes that exhibited significant differences between the CS and SB groups. To further analyze the impact of 3D clinostat on the brain, we conducted KEGG pathway analysis of the differentially expressed genes, as shown in Fig. 4 c. 3D clinostat may primarily affect immune and endocrine-related signaling pathways, such as those associated with human diseases like Malaria, African trypanosomiasis, Autoimmune thyroid disease, Rheumatoid arthritis, and Staphylococcus aureus infection, as well as GnRH secretion and signaling pathway, Ovarian steroidogenesis, IL17 signaling pathway, Prolactin signaling pathway, Leukocyte transendothelial migration, PPAR signaling pathway, and Neutrophil extracellular trap formation.

### The impact of 3D clinostat on serum metabolomics

To investigate the systemic effects of the 3D clinostat on mice, we conducted a non-targeted metabolomics analysis of serum samples. This experiment yielded a total of 15,470 detected substances, among which 1431 were annotated as known metabolic molecules. OPLS-DA analysis revealed distinct differentiation among the three groups (Fig. 4d). Notably, in comparison to the SB group, the CS group exhibited significant upregulation of serum metabolites such as Lys-Leu, 2-keto-D-Gluconic acid, Thyroxine 4’-o-.beta.-d-glucuronide, Beta-octylglucoside, Demissidine, Pro-Trp, and Pentobarbital. Conversely, serum metabolites including Poricoic acid a, L-Lysine, Oleic acid, Ile-Pro, 1,2-dioleoyl-sn-glycero-3-phosphoethanolamine-n,n-dimethyl, Niacinamide, and 1-(1z-octadecenyl)-2-(4z,7z,10z,13z,16z,19z-docosahexaenoyl)-sn-glycero-3-phosphocholine were significantly downregulated (Fig. 4e). These alterations primarily implicate pathways such as ABC transporters, Sphingolipid signaling pathway and metabolism, Biotin metabolism, and the Pentose phosphate pathway (Fig. 4f).

### The impact of 3D clinostat on microbiome

**Fig. 5:**
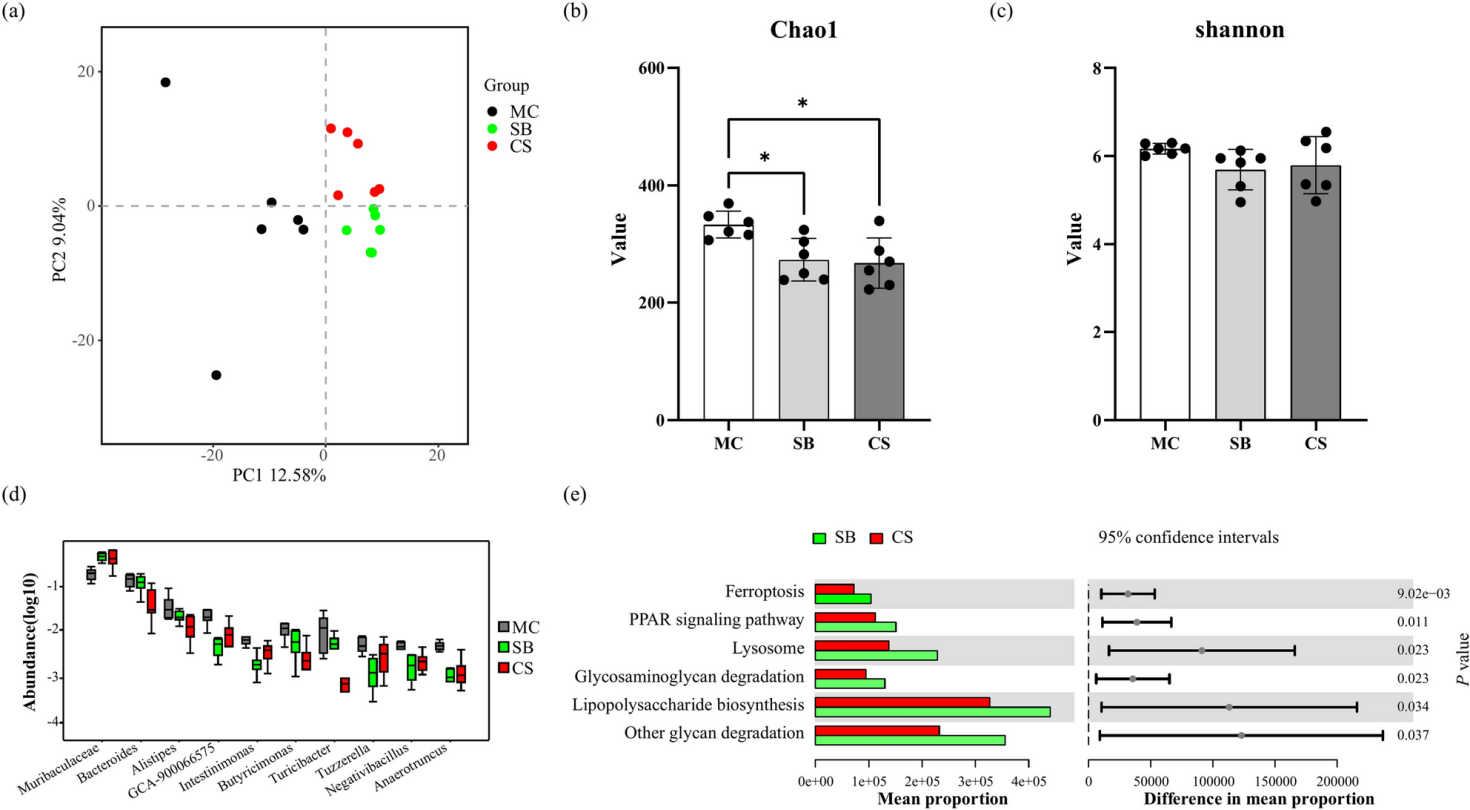
Changes in the fecal microbiome of of mice subjected to 3D clinostat. (**a**) PCA analysis of the fecal microbiome among the three groups of mice; (**b**) Alpha diversity analysis using Chao1 index; (**c**) Alpha diversity analysis using Shannon index; (**d**) Changes in the top 10 differentially abundant bacteria at the species level; (**e**) KEGG functional analysis of the microbial community differences between the CS and SB groups. MC: ordinary cage control group; SB: survival box control group; CS: 3D clinostat model group. The data are presented as the mean ± SD, *P < 0.05, n = 6 for each group.

**Fig. 6:**
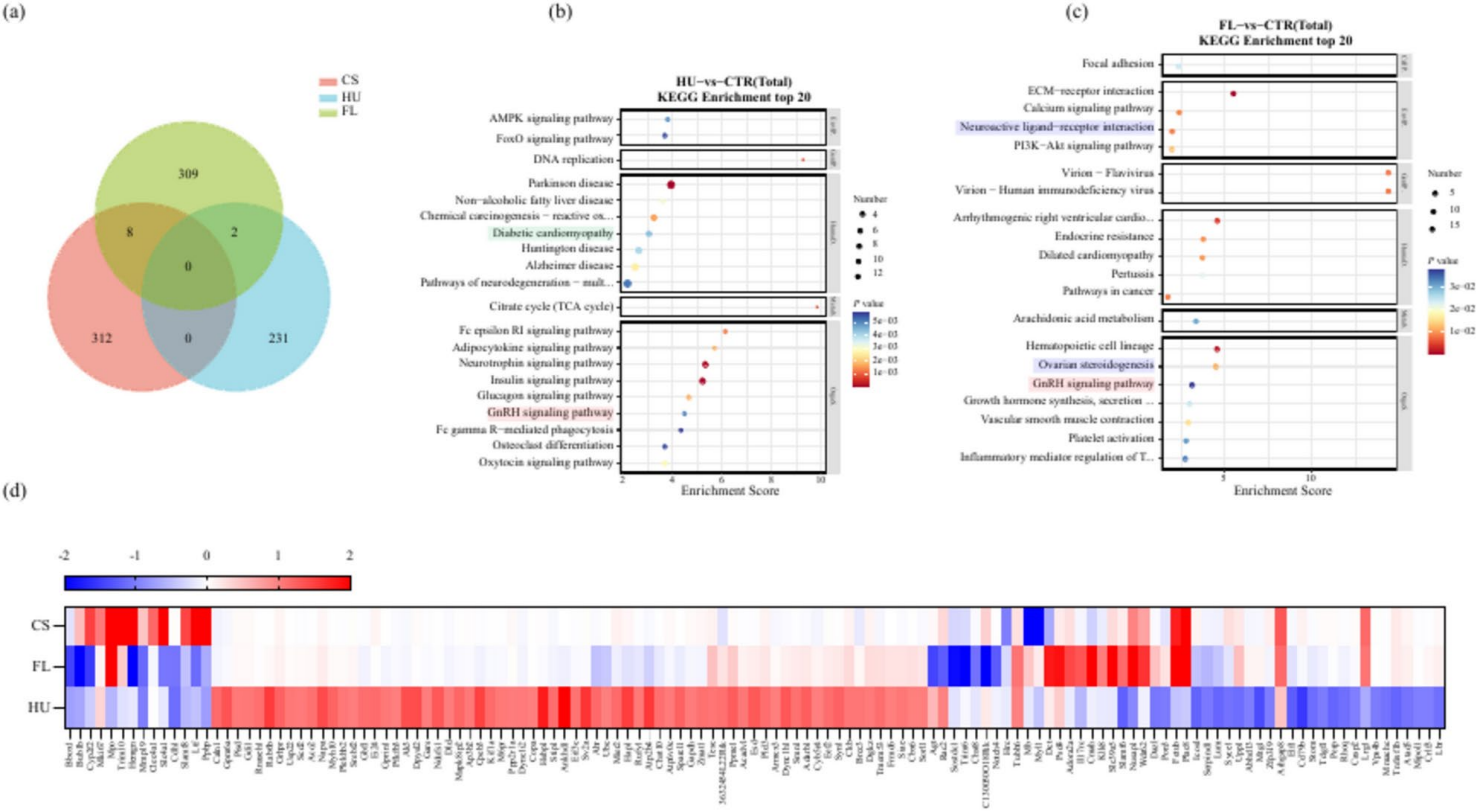
Comparison of the 3D clinostat simulated microgravity effect model with the classical hindlimb unloading model and spaceflight mice. (**a**) Venn diagram of differentially expressed genes in brain transcriptomes among the three models; (**b**) Main KEGG pathways affected in the hindlimb unloading model; (**c**) Main KEGG pathways affected in the spaceflight model; (**d**) Heatmap visualizing the expression levels of 131 genes commonly detected in all three models and fulfilling the criteria of differential expression (*P* value < 0.05 and fold change > 2 or fold change < 0.5) in at least one model. Red markers: KEGG pathways commonly affected by all three models; Green markers: KEGG pathways commonly affected by the HU and FL models; Blue markers: KEGG pathways commonly affected by the CS and FL models. CS: 3D clinostat model; HU: hindlimb unloading model; FL: spaceflight model.

To investigate the impact of 3D clinostat on the gastrointestinal microbiota, 16S rRNA sequencing was performed on mouse feces samples, revealing 333 ± 23, 273 ± 36, and 267 ± 43 ASVs detected in each group, respectively. In terms of alpha diversity, the fecal microbiota richness was observed to be higher in the MC group compared to the SB and CS groups (Fig. 5b). However, no significant differences were observed in the Shannon index among the three groups (Fig. 5c). Regarding beta diversity, PCA analysis demonstrated distinct segregation of the fecal microbiota profiles among the three groups (Fig. 5a). At the genus level, Fig. 6d illustrated the top 10 differentially abundant bacterial genera among the three groups. Notably, the *Bacteroides* and *Turicibacter* were significantly reduced in the CS group compared to both the SB and MC groups (Fig. 5d). Functional analysis of the microbial community was conducted using the PICRUSt2 software. The results indicated significant differences in KEGG signaling pathways between the CS and SB groups, specifically in Ferroptosis, PPAR signaling pathway, Lysosome, Glycosaminoglycan degradation, Lipopolysaccharide biosynthesis, and Other glycan degradation (Fig. 5e). These findings provide valuable insights into the effects of 3D clinostat on the composition and functional properties of the gastrointestinal microbiota, suggesting potential implications for host health.

### Comparison of 3D clinostat simulated microgravity effect model with classical hindlimb unloading model and spaceflight mice.

To comprehensively assess the validity of the 3D clinostat simulated microgravity effect model, we conducted a comparative analysis of mRNA expression profiles in brain tissue, juxtaposing our findings with those derived from literature on hindlimb unloading (HU) mice and spaceflight (FL) mice models. The study revealed minimal overlap among the three models, underscoring substantial variations. Specifically, eight differentially expressed genes (Ibsp, Adam3, Xlr3b, G530011O06Rik, Wdr62, Cd22, Gm4675, and Nusap1) were shared between the CS and FL models, whereas only two genes (Serpinc1 and Ccl19) overlapped between the HU and FL models. Notably, no shared differentially expressed genes were identified between the CS and HU models, highlighting notable disparities between these two models (Fig. 6a). These findings imply that neither the CS nor the HU model perfectly recapitulates the complex effects of actual space microgravity on the brain, albeit the CS model demonstrates relatively greater fidelity compared to the HU model.

With respect to the functional disparities among genes, the HU model predominantly features genes tied to neurodegenerative disorders, whereas the FL model encompassed a diverse array of genes related to immunity, endocrinology, cardiovascular functions, and beyond. Among the top 20 KEGG pathways, the GnRH signaling pathway stands out as a significantly altered pathway across all three models, highlighted in red. Notably, the CS and FL models share additional two commonly altered pathways: Neuroactive ligand-receptor interaction and Ovarian steroidogenesis, both denoted with blue. Furthermore, Diabetic cardiomyopathy, indicated with green, emerges as a shared altered pathway between the CS and HU models. These observations imply that the CS model exhibited a greater degree of similarity to the FL model in comparison to the HU model (Fig. 6b–c).

To compare the differences in brain gene expression profiles across these three models, we performed a comprehensive distance comparison involving 5687 genes that were consistently detected in all models. The results of this analysis are as follows: S_CS/FL_ = $\sum_{1}^{n}\left|{FC}_{cs}n-{FC}_{FL}n\right|=747.25$, S_HU/FL_ = $\sum_{1}^{n}|{FC}_{HU}n-{FC}_{FL}n|=1756.26$, and S_CS/HU_ = $\sum_{1}^{n}|{FC}_{cs}n-{FC}_{HU}n|=1575.88$. Notably, the S_CS/FL_ distance emerges as significantly smaller than the S_HU/FL_ distance, underscoring the closer resemblance of the CS model to the FL model in terms of gene expression patterns. Additionally, by generating a heatmap that visualizes the expression levels of 131 genes commonly detected in all three models and fulfilling the criteria of differential expression (*P* value < 0.05 and and foldchange > 2 or foldchange < 0.5) in at least one model, it becomes strikingly apparent that the CS model demonstrates a superior performance compared to the HU model (Fig. 6d).

## Discussion

This study explored the impact of simulated microgravity effect using a 3D clinostat on mice, with a particular focus on behavioral, metabolic, gut microbiome and brain transcriptome changes. Our findings revealed that the simulated microgravity effect induced by the 3D clinostat triggered noteworthy transformations within the central nervous system. This model has the potential to serve as a valuable tool for investigating cognitive changes associated with microgravity environments, while also offering new insights into the potential mechanisms underlying spaceflight-related health risks.

In this study, the behavioral experiments demonstrated that the 3D clinostat had a significant impact on the physical activity and exploratory behavior. The observed reduction in movement distance and exercise time in the open field test, combined with similar findings in the Y Maze, elevated plus maze, and novel object recognition tests, suggests a marked decrease in the overall motivation and exploratory drive. These results are consistent with existing literature on the effects of microgravity, where diminished activity levels and altered behavioral patterns have been reported^19–21^. For instance, mice displayed similar declines in exploratory behavior after a 30-day spaceflight on Bion-M1 satellite^21^. Different from past results, we did not find obvious anxiety or depression in mice^20,22,23^. It is known that chronic restraint can lead to anxiety and depression^24^. We speculate that the anxiety or depression emotion of animals found in the HU model might be caused by tail suspension and restraint, rather than the direct effect of microgravity.

The absence of notable disparities in learning and memory between the CS and SB groups, as assessed by the Morris water maze and other memory related tests, might initially seem surprising. One would expect that the reduced physical activity and altered emotional states might extend to cognitive functions. However, the results suggest that the impact of microgravity on cognition might be more complex, potentially involving compensatory mechanisms that preserve cognitive abilities despite changes in other behavioral domains. This finding aligns with studies in which astronauts, despite experiencing emotional and physical stressors, often maintain cognitive performance during space missions, possibly due to intensive training and adaptation processes^25^. In fact, there are indeed some controversies about the effects of microgravity on cognitive function^25–27^. During spaceflights, the overall cognitive performance of astronauts does not show significant decline, but their cognitive function declines notably after returning to Earth^25^. Wollseiffen et al. found that neurocognitive performance can even be improved during short-term microgravity, thus suspecting that cognitive impairment is caused by the combined effects of complex spaceflight stressors rather than microgravity itself^28^. The adaptive changes such as changes in brain blood supply caused by re-adaptation to the gravitational environment after the flight, rather than microgravity itself, may be the key factors that lead to changes in cognitive function. Besides, it is also possible that the short duration of microgravity effect exposure in our study was insufficient to produce detectable cognitive deficits. Future studies with prolonged exposure and more sensitive cognitive assessments might reveal subtle impairments not captured in this study.

The motor coordination and balance tests, such as the rotarod and gait analysis, indicated subtle yet significant changes in the vestibular system of the mice. These alterations are particularly relevant given the well-documented vestibular dysfunctions reported by astronauts during and after space missions^29^. The role of vestibular system in spatial orientation and balance is crucial, and its disruption in microgravity can lead to disorientation, dizziness, and impaired motor function^30^. Under the premise that there was no significant change in grip strength, our findings indicated that the latency to fall in the rotarod test for CS group was increased compared to SB group, suggesting an adaptation or learning process that may mimic the vestibular compensation observed in astronauts. This adaptive response could be due to the continuous 3D clinostat environment, which might induce a reorganization of neural circuits involved in balance and coordination.

Interestingly, the fear conditioning test showed that the 3D clinostat treated mice exhibited an increased sensitivity to fear stimuli and enhanced fear memory. This observation might be linked to changes in the amygdala and related brain structures, which are critical for processing fear and stress responses^31^. Research on rats has revealed significant alterations in amygdala neural activity after landing, marked by Fos-positive cells, suggesting a potential impact on fear-related emotional responses^32,33^. In 15 days of –6° head-down bed rest, volunteers showed temporal overestimation of the fear stimuli in the middle phase^34^. This finding has important implications for understanding the psychological and emotional challenges faced by astronauts during long-term space missions, where prolonged exposure to microgravity could exacerbate stress and anxiety, potentially impairing mission performance and well-being. In the conditioned fear experiment, the most significant difference between the CS and SB groups occurred during the fear extinction phase, suggesting an attenuation of fear memory extinction. The specific neural circuit mechanisms underlying this phenomenon require further in-depth research.

In terms of bone density, mice in the CS group exhibited a decrease in bone volume and trabecular number, accompanied by a notable increase in trabecular pattern factor and trabecular separation, indicating bone loss changes, when compared to the MC control group. However, despite a seemingly more pronounced tendency towards bone loss in the CS group compared to the SB group, no statistically significant difference was observed. This may be attributed to the enhanced gripping and limb loading during the 3D clinostat process, as the mice attempted to counteract the body repositioning caused by the rotation. In the rotarod test, the prolonged fall latency observed in mice from the CS group, as well as subtle gait alterations detected in gait analysis, may also be associated with the similar underlying reasons. On the other hand, the restricted space within the survival box likely constrained the motor abilities of the SB group mice, and simultaneously induced bone loss in these mice. This could potentially account for the reduced difference between the CS and SB groups, representing another plausible explanation.

The transcriptomic analysis of the brain tissues revealed significant changes in gene expression associated with immune response, endocrine signaling, and nervous system functions. These alterations suggested that the simulated microgravity effect triggered a broad and complex biological response, affecting multiple systems that are crucial for maintaining homeostasis and health. These findings are consistent with previous studies that have reported immune dysregulation and hormonal imbalances in response to microgravity in spaceflight animal models^35,36^. Immune dysregulation and hormonal imbalances could lead to a range of health problems, including increased susceptibility to infections, metabolic disorders, and mental health issues. Effective monitoring and the development of targeted interventions could help mitigate these risks and improve the health of astronauts.

One of the key findings of this study is the differential gene expression pattern observed in the CS model compared to the HU and FL models. The minimal overlap in differentially expressed genes between these models highlights the distinct biological effects induced by each type of simulated microgravity effect. Specifically, the CS model appears to more closely mimic the gene expression changes seen in real spaceflight, particularly in terms of immune and endocrine-related pathways. This suggests that the 3D clinostat might be a more accurate and reliable model for studying the effects of microgravity on the central nervous system. The CS and FL models demonstrated a convergent alteration in three KEGG signaling pathways: GnRH signaling pathway, Neuroactive ligand-receptor interaction, and Ovarian steroidogenesis, as well as eight common differential genes, including Ibsp, Adam3, Xlr3b, G530011O06Rik, Wdr62, Cd22, Gm4675, and Nusap1. The GnRH signaling pathway plays a pivotal role in the hypothalamus, regulating the function of the hypothalamic–pituitary–gonadal (HPG) axis. Under microgravity conditions, significant changes occur in the endocrine system, which may impact the release of GnRH and the activity of the signaling pathway. This alteration in the GnRH signaling pathway may subsequently lead to fluctuations in sex hormone levels and participate in the regulation of cognition, emotion, and behavior^37^. The Neuroactive Ligand-Receptor Interaction pathway encompasses the intricate interplay between numerous neurotransmitters and hormones (such as dopamine, serotonin, GABA, glutamate, and others) and their respective receptors. This interaction is vital for neural transmission, emotional regulation, cognitive functions, and neural plasticity. Microgravity and spaceflight can potentially induce alterations in neural adaptability, as well as changes in the levels and sensitivity of neurotransmitters, which may be linked to the Neuroactive Ligand-Receptor Interaction^38^. The Ovarian Steroidogenesis signaling pathway is concerned with the production of estrogen and progesterone. Although ovarian steroidogenesis primarily occurs within the reproductive system, it also exerts significant influence on the brain, particularly in regulating emotions, cognition, and neural plasticity^39^. Furthermore, estrogen possesses neuroprotective properties, contributing to the maintenance of neuronal health and function^40^. Under microgravity conditions, a decrease in estrogen levels may diminish its neuroprotective effects, potentially increasing the risk of neurodegenerative diseases or cognitive decline. The greater similarity in gene expression profiles between the CS and FL models supports the use of this method as a representative model for spaceflight-induced neurobiological changes.

The lack of overlap in differentially expressed genes between the HU and FL models further underscores the limitations of the HU model in replicating the effects of actual spaceflight. While the HU model has been widely used to simulate microgravity effect, particularly for studying muscle atrophy and bone loss, it appears to fall short in accurately modeling the complex changes in brain function induced by real spaceflight^41,42^. This discrepancy may be due to the localized nature of the HU model, which primarily affects the hindlimbs and may not adequately capture the whole-body effects of microgravity. In contrast, the 3D clinostat model provides a more holistic simulation of microgravity effect, potentially leading to more relevant insights into how spaceflight affects the brain. The HU model demonstrates a heightened sensitivity to alterations in cerebral blood flow, rendering it an appropriate choice for investigating notable cognitive shifts following return from spaceflight, while the 3D clinostat model is more likely to focus on subtle cognitive changes caused by adaptation to microgravity in space, which may be related to vestibular function and spatial orientation.

The metabolomic analysis of serum samples provided additional insights into the systemic effects of 3D clinostat model. The observed alterations in various metabolic pathways, including ABC transporters, Sphingolipid signaling, and the Pentose phosphate pathway, suggest that microgravity effect disrupts lipid metabolism, energy production, and cellular signaling processes. The downregulation of key metabolites such as L-Lysine, oleic acid, and niacinamide points to potential deficiencies in essential nutrients, which could exacerbate the stress response and impair overall health. L-Lysine is an essential amino acid involved in protein synthesis, while oleic acid is a major component of membrane phospholipids and has anti-inflammatory properties^43^. Niacinamide, a form of vitamin B3, is crucial for energy production and DNA repair^44,45^. The reduction in these metabolites suggests that microgravity might lead to nutritional imbalances that could have far-reaching effects on health.

The microbiome analysis further supports these findings, showing significant alterations in gut microbiota composition, particularly the reduction in *Bacteroides* and *Turicibacter*. The reduction in *Bacteroides* is consistent with the reported elevation of the *Firmicutes*/*Bacteroidetes* ratio in the intestinal flora of astronauts during spaceflights^25^. The gut microbiota plays a critical role in regulating immune function, metabolism, and overall health, and its disruption could have serious consequences during spaceflight. Previous studies have shown that changes in the gut microbiota can lead to a range of health issues, including immune dysregulation, metabolic disorders, and even mental health problems^45^. The observed changes in the gut microbiota in this study suggest that simulated microgravity effect may lead to similar health challenges, emphasizing the need for strategies to maintain gut health during long-term space missions. The functional analysis of the microbial community revealed significant differences in KEGG signaling pathways between the CS and SB groups. Pathways such as Ferroptosis, PPAR signaling, and Glycosaminoglycan degradation were notably affected, indicating potential alterations in cellular death processes, lipid metabolism, and extracellular matrix remodeling. These changes could have implications for tissue integrity and function, particularly in the gastrointestinal tract, which is highly sensitive to environmental stressors^46^. The disruption of these pathways could contribute to the gastrointestinal problems of astronauts, such as increased gut permeability, altered bowel habits and increased susceptibility to infections^47^. The alterations in gut microbiota composition observed in this study also suggest that dietary interventions or probiotics could be explored as potential strategies to maintain gut health during space missions.

The findings of this study have significant implications for understanding the health risks associated with long-term spaceflight. The 3D clinostat model offers a practical and cost-effective method for simulating microgravity effect on Earth, allowing for the systematic study of its effects on various physiological systems. The results suggest that microgravity may have a more pronounced impact on emotional and motivational states than on cognitive functions, highlighting the need for further research into the psychological well-being of astronauts during extended missions.

While our study provided important insights into the 3D clinostat simulated microgravity effect, it is essential to acknowledge certain limitations. Firstly, although some behavioral changes were observed, many of them did not reach statistical significance, potentially due to the relatively small sample size in the study, leading to the possibility that subtle behavioral changes might have been overlooked. Secondly, in space environments, males and females exhibit gender-specific differences in the responses of various bodily systems. This study exclusively used male mice; however, considering the impact of gender factors, it is a scientifically valuable question worth further investigation to determine whether female mice exhibit different responses to the 3D clinostat. Furthermore, we noted significant differences in multiple behavioral indicators, as well as in the transcriptome and metabolome, between mice in the survival box control (SB) group compared to ordinary cage control (MC) group, highlighting the crucial impact of social isolation and spatial confinement on the organism. Further analysis of the synergistic effects of microgravity, social isolation, and spatial constraints is a scientific issue worthy of further attention in the next step. Moreover, it is important to acknowledge that variability in transcriptome data may be influenced by differences in time points, exposure durations, and collection methodologies employed across clinostat studies, hindlimb unloading experiments, and spaceflight missions. Additionally, although mice spend most of their time inactive in the resting chamber of the survival box, they may still be forced to move or perform actions against gravity-direction changes. It is acknowledged that the 3D clinostat and hindlimb unloading models do not truly simulate microgravity in a strict physical sense. The 3D clinostat creates a condition where the gravity orientation continuously changes, and the hindlimb unloading model provides mechanical unloading. However, our study focuses on the biological effects that resemble those under microgravity conditions. The data from the 3D clinostat—treated mice show changes in behavior, metabolism, microbiome, and brain transcriptome. Although these changes may be due to the mice’s response to altered gravity orientation rather than actual microgravity, they share similarities with the biological effects observed in spaceflight studies. For instance, the changes in the vestibular system, as indicated by gait analysis, are consistent with the vestibular dysfunctions reported by astronauts during space missions. Similarly, the alterations in immune and endocrine—related signaling pathways in the brain transcriptome are also in line with previous spaceflight—related research. These similarities suggest that the 3D clinostat model can serve as a valuable tool for studying the potential biological consequences of microgravity on Earth. It is important to acknowledge the differences in physical principles between our models and actual spaceflight, and when selecting models, one must be mindful of these differences to ensure that the appropriate animal model is chosen according to the research objectives. Nevertheless, due to the limitations and difficulties of simulating microgravity on Earth, the 3D clinostat mouse model for simulating microgravity is a powerful supplement to the limited ground-based models currently available. The 3D clinostat model provided a more similar simulation of brain gene expression alterations than hindlimb unloading, yet future research is essential to investigate the underlying mechanisms of the observed behavioral and neurobiological changes, particularly through targeted studies on specific brain regions and molecular pathways. For example, studying the effects of microgravity effect on the hippocampus, amygdala, and prefrontal cortex could provide deeper insights into how microgravity impacts cognitive function and emotional regulation. Additionally, exploring potential therapeutic interventions could provide valuable insights into mitigating the adverse effects of microgravity on astronaut health. Pharmacological treatments that target specific pathways affected by microgravity, such as immune or endocrine signaling, could also help maintain health during long-term space missions.

## Conclusion

In summary, our research underscored that 3D clinostat model exerted profound effects on the behavior, metabolism, microbiome, and brain transcriptome. Compared to hindlimb unloading model, the 3D clinostat model demonstrates a closer resemblance to the brain transcriptome expression profiles observed in spaceflight animals, thereby offering a novel and invaluable research tool for simulating microgravity effect on Earth.

## Supplementary Information

Supplementary Information.

## Data Availability

The data that support the findings of this study have been deposited into CNGB Sequence Archive (CNSA) of China National GeneBank DataBase (CNGBdb) with accession number CNP0006448 (https://db.cngb.org/search/project/CNP0006448). Additionally, the corresponding author is available to provide data upon request (guojianguo@cnilas.org).
