# Supplementary material for: Behavioral and multiomics analysis of 3D clinostat simulated microgravity effect in mice focusing on the central nervous system
Source: Sci Rep. 2025 Feb 17;15:5731. doi: 10.1038/s41598-025-90212-y (PMC11833055; doi:10.1038/s41598-025-90212-y)
Supplement: Supplementary file 1 — Supplementary Information. [file 41598_2025_90212_MOESM1_ESM.docx]

1. Supplementary Table S1：Gait analysis raw data

| Trial_Description | MC1 | MC2 | MC3 | MC4 | MC5 | MC6 | SB1 | SB2 | SB3 | SB4 | SB5 | SB6 | CS1 | CS2 | CS3 | CS4 | CS5 | CS6 | MC  (Mean±SD) | SB  (Mean±SD) | CS  (Mean±SD) |
| --- | --- | --- | --- | --- | --- | --- | --- | --- | --- | --- | --- | --- | --- | --- | --- | --- | --- | --- | --- | --- | --- |
| Run_Average_Speed_(cm/s) | 33.69 | 24.12 | 38.53 | 27.65 | 23.83 | 52.86 | 26.05 | 25.97 | 28.37 | 44.26 | 31.65 | 40.30 | 35.95 | 52.81 | 17.90 | 35.10 | 23.20 | 28.74 | **33.45±11.1** | **32.77±7.76** | **32.28±12.2** |
| RF_PrintLength_(cm)_Mean | 0.81 | 0.88 | 0.76 | 0.91 | 0.82 | 0.83 | 0.89 | 0.91 | 0.91 | 0.95 | 0.76 | 0.79 | 0.86 | 0.75 | 0.74 | 0.70 | 0.74 | 0.75 | **0.84±0.05** | **0.87±0.08** | **0.75±0.05** |
| RF_PrintWidth_(cm)_Mean | 0.81 | 0.69 | 0.57 | 0.75 | 0.74 | 0.73 | 0.72 | 0.77 | 0.81 | 0.74 | 0.53 | 0.80 | 0.64 | 0.67 | 0.60 | 0.59 | 0.56 | 0.62 | **0.72±0.08** | **0.73±0.1** | **0.61±0.04** |
| RF_PrintArea_(cm²)_Mean | 0.25 | 0.31 | 0.20 | 0.34 | 0.29 | 0.24 | 0.34 | 0.36 | 0.38 | 0.37 | 0.25 | 0.29 | 0.31 | 0.24 | 0.25 | 0.21 | 0.21 | 0.27 | **0.27±0.05** | **0.33±0.05** | **0.25±0.04** |
| RH_MaxContactAt_(%)_Mean | 32.99 | 30.56 | 30.08 | 33.19 | 31.74 | 32.86 | 43.58 | 27.84 | 29.68 | 28.79 | 35.51 | 29.89 | 35.62 | 34.62 | 42.83 | 37.35 | 39.65 | 38.92 | **31.9±1.34** | **32.55±6.03** | **38.17±2.97** |
| RH_MaxContactArea_(cm²)_Mean | 0.26 | 0.26 | 0.20 | 0.31 | 0.25 | 0.19 | 0.24 | 0.27 | 0.21 | 0.20 | 0.22 | 0.27 | 0.24 | 0.19 | 0.24 | 0.17 | 0.19 | 0.21 | **0.24±0.04** | **0.23±0.03** | **0.21±0.03** |
| RH_MaxContactMaxIntensity_Mean | 191.98 | 187.36 | 193.25 | 197.98 | 180.64 | 181.41 | 188.69 | 177.35 | 177.87 | 166.34 | 192.26 | 196.25 | 166.26 | 172.82 | 183.52 | 140.94 | 148.75 | 155.42 | **188.77±6.89** | **183.13±11.22** | **161.28±15.86** |
| RH_MaxContactMeanIntensity_Mean | 82.65 | 83.83 | 86.63 | 84.14 | 79.88 | 85.17 | 80.18 | 75.67 | 82.97 | 77.80 | 85.82 | 84.74 | 77.37 | 80.33 | 81.64 | 70.85 | 73.08 | 73.52 | **83.72±2.31** | **81.2±4.01** | **76.13±4.32** |
| RH_PrintLength_(cm)_Mean | 0.84 | 0.90 | 0.78 | 0.92 | 0.85 | 0.75 | 0.87 | 1.04 | 0.87 | 0.87 | 0.90 | 0.85 | 0.88 | 0.80 | 0.84 | 0.73 | 0.78 | 0.87 | **0.84±0.07** | **0.9±0.07** | **0.82±0.06** |
| RH_PrintWidth_(cm)_Mean | 0.63 | 0.67 | 0.70 | 0.75 | 0.61 | 0.58 | 0.75 | 0.72 | 0.67 | 0.65 | 0.70 | 0.64 | 0.64 | 0.62 | 0.64 | 0.65 | 0.57 | 0.60 | **0.66±0.06** | **0.69±0.04** | **0.62±0.03** |
| RH_PrintArea_(cm²)_Mean | 0.29 | 0.30 | 0.23 | 0.36 | 0.29 | 0.21 | 0.30 | 0.32 | 0.26 | 0.24 | 0.28 | 0.30 | 0.29 | 0.24 | 0.30 | 0.20 | 0.23 | 0.25 | **0.28±0.05** | **0.28±0.03** | **0.25±0.04** |
| RH_MaxIntensityAt_(%)_Mean | 35.37 | 53.45 | 43.31 | 54.51 | 54.64 | 43.13 | 54.40 | 52.77 | 54.68 | 62.43 | 56.93 | 38.27 | 42.12 | 38.46 | 44.86 | 54.01 | 45.37 | 45.67 | **47.4±7.99** | **53.25±8.07** | **45.08±5.15** |
| RH_MaxIntensity_Mean | 195.13 | 192.81 | 196.48 | 202.14 | 189.37 | 186.57 | 192.30 | 186.76 | 188.20 | 178.05 | 198.64 | 198.89 | 171.22 | 177.86 | 188.60 | 148.14 | 155.83 | 162.31 | **193.75±5.5** | **190.47±7.93** | **167.33±14.86** |
| RH_MinIntensity_Mean | 35.77 | 36.32 | 37.06 | 35.71 | 35.44 | 37.37 | 35.41 | 35.40 | 35.80 | 38.03 | 35.67 | 36.14 | 36.50 | 36.67 | 35.89 | 36.93 | 35.88 | 35.92 | **36.28±0.78** | **36.07±0.99** | **36.3±0.46** |
| RH_MeanIntensity_Mean | 89.61 | 88.14 | 89.94 | 90.73 | 86.67 | 90.71 | 84.65 | 79.71 | 86.90 | 79.72 | 90.42 | 91.64 | 81.56 | 82.03 | 85.77 | 75.55 | 78.64 | 77.43 | **89.3±1.6** | **85.5±5.13** | **80.16±3.69** |
| RH_MeanIntensityOfThe15MostIntensePixels_Mean | 143.53 | 142.64 | 136.45 | 152.77 | 136.38 | 129.08 | 137.71 | 127.07 | 134.24 | 112.06 | 142.41 | 149.01 | 119.32 | 118.51 | 138.38 | 102.21 | 113.11 | 112.03 | **140.14±8.09** | **133.75±12.95** | **117.26±12.02** |
| RH_InitialDualStance_(s)_Mean | 0.01 | 0.01 | 0.01 | 0.03 | 0.01 | 0.00 | 0.01 | 0.02 | 0.01 | 0.03 | 0.01 | 0.01 | 0.03 | 0.02 | 0.03 | 0.02 | 0.01 | 0.05 | **0.011±0.011** | **0.013±0.007** | **0.025±0.012** |
| RH_TerminalDualStance_(s)_Mean | 0.02 | 0.02 | 0.01 | 0.03 | 0.01 | 0.01 | 0.01 | 0.03 | 0.01 | 0.02 | 0.01 | 0.02 | 0.03 | 0.02 | 0.03 | 0.02 | 0.02 | 0.05 | **0.015±0.007** | **0.017±0.008** | **0.028±0.011** |
| LF_MaxContactArea_(cm²)_Mean | 0.26 | 0.24 | 0.24 | 0.31 | 0.28 | 0.29 | 0.27 | 0.27 | 0.31 | 0.32 | 0.23 | 0.29 | 0.24 | 0.22 | 0.17 | 0.18 | 0.16 | 0.24 | **0.27±0.03** | **0.28±0.03** | **0.2±0.04** |
| LF_MaxContactMaxIntensity_Mean | 159.01 | 162.58 | 161.90 | 179.18 | 166.53 | 165.09 | 163.34 | 166.25 | 172.53 | 161.32 | 149.77 | 167.24 | 154.38 | 145.03 | 122.02 | 140.10 | 133.99 | 155.24 | **165.72±7.09** | **163.41±7.7** | **141.79±12.68** |
| LF_MaxContactMeanIntensity_Mean | 71.17 | 70.34 | 71.28 | 78.85 | 74.91 | 74.31 | 72.76 | 73.93 | 75.35 | 77.38 | 71.81 | 74.97 | 72.52 | 70.06 | 64.32 | 66.00 | 65.44 | 73.82 | **73.48±3.21** | **74.37±1.99** | **68.69±3.99** |
| LF_PrintLength_(cm)_Mean | 0.91 | 0.93 | 0.82 | 0.90 | 0.86 | 0.91 | 0.92 | 0.89 | 0.95 | 0.93 | 0.75 | 0.96 | 0.79 | 0.76 | 0.70 | 0.65 | 0.78 | 0.90 | **0.89±0.04** | **0.9±0.08** | **0.76±0.09** |
| LF_PrintWidth_(cm)_Mean | 0.77 | 0.77 | 0.76 | 0.76 | 0.71 | 0.76 | 0.73 | 0.70 | 0.82 | 0.75 | 0.63 | 0.72 | 0.70 | 0.64 | 0.59 | 0.59 | 0.64 | 0.70 | **0.75±0.02** | **0.72±0.06** | **0.64±0.05** |
| LF_PrintArea_(cm²)_Mean | 0.33 | 0.32 | 0.30 | 0.37 | 0.33 | 0.33 | 0.34 | 0.34 | 0.37 | 0.37 | 0.27 | 0.36 | 0.28 | 0.24 | 0.20 | 0.21 | 0.22 | 0.31 | **0.33±0.02** | **0.34±0.04** | **0.24±0.04** |
| LF_MaxIntensityAt_(%)_Mean | 63.57 | 59.29 | 61.05 | 54.50 | 59.97 | 55.00 | 60.32 | 63.50 | 61.07 | 57.67 | 58.80 | 50.84 | 57.40 | 47.61 | 67.80 | 59.84 | 67.70 | 59.01 | **58.9±3.53** | **58.7±4.34** | **59.89±7.5** |
| LF_MaxIntensity_Mean | 177.73 | 171.67 | 169.36 | 184.93 | 175.20 | 170.63 | 170.63 | 177.39 | 180.69 | 170.34 | 156.33 | 177.54 | 159.56 | 151.34 | 131.54 | 145.53 | 146.08 | 166.43 | **174.92±5.8** | **172.15±8.78** | **150.08±12.16** |
| LF_MinIntensity_Mean | 36.48 | 36.01 | 35.96 | 35.76 | 35.54 | 36.18 | 35.37 | 36.20 | 35.66 | 36.63 | 36.88 | 36.53 | 36.65 | 37.52 | 35.94 | 36.93 | 36.64 | 36.29 | **35.99±0.32** | **36.21±0.59** | **36.66±0.54** |
| LF_MeanIntensity_Mean | 73.91 | 74.50 | 73.64 | 82.93 | 78.74 | 77.06 | 75.89 | 76.74 | 79.29 | 81.14 | 75.08 | 79.16 | 75.14 | 74.20 | 68.50 | 68.95 | 69.39 | 77.31 | **76.8±3.61** | **77.88±2.34** | **72.25±3.76** |
| LF_MeanIntensityOfThe15MostIntensePixels_Mean | 116.86 | 116.37 | 113.66 | 134.77 | 124.40 | 119.08 | 120.16 | 122.58 | 125.40 | 128.09 | 112.92 | 126.92 | 111.24 | 103.85 | 89.24 | 92.44 | 94.50 | 117.35 | **120.86±7.71** | **122.68±5.59** | **101.44±11.25** |
| LH_MaxContactAt_(%)_Mean | 30.50 | 34.02 | 20.12 | 29.48 | 29.26 | 34.47 | 31.56 | 33.37 | 26.36 | 41.03 | 37.85 | 29.37 | 45.39 | 41.81 | 51.97 | 39.04 | 46.14 | 39.99 | **29.64±5.18** | **33.26±5.42** | **44.06±4.8** |
| LH_MaxContactArea_(cm²)_Mean | 0.24 | 0.22 | 0.22 | 0.31 | 0.25 | 0.24 | 0.24 | 0.24 | 0.24 | 0.21 | 0.22 | 0.26 | 0.21 | 0.24 | 0.21 | 0.19 | 0.16 | 0.21 | **0.25±0.03** | **0.23±0.02** | **0.21±0.03** |
| LH_MaxContactMaxIntensity_Mean | 191.45 | 188.06 | 173.28 | 198.83 | 173.96 | 177.87 | 186.97 | 197.90 | 165.93 | 172.38 | 190.52 | 190.17 | 146.97 | 154.80 | 178.18 | 169.21 | 165.78 | 162.23 | **183.91±10.44** | **183.98±12.2** | **162.86±10.97** |
| LH_MaxContactMeanIntensity_Mean | 79.06 | 81.77 | 75.65 | 83.58 | 80.85 | 79.35 | 77.93 | 84.58 | 77.17 | 75.19 | 84.76 | 83.54 | 72.97 | 73.58 | 80.73 | 74.35 | 79.10 | 76.32 | **80.04±2.72** | **80.53±4.24** | **76.17±3.15** |
| LH_PrintLength_(cm)_Mean | 0.80 | 0.86 | 0.83 | 0.89 | 0.85 | 0.82 | 0.98 | 0.99 | 0.94 | 0.85 | 0.86 | 0.83 | 0.81 | 0.89 | 0.86 | 0.85 | 0.78 | 0.80 | **0.84±0.03** | **0.91±0.07** | **0.83±0.04** |
| LH_PrintWidth_(cm)_Mean | 0.67 | 0.59 | 0.62 | 0.75 | 0.67 | 0.65 | 0.74 | 0.71 | 0.64 | 0.60 | 0.62 | 0.59 | 0.59 | 0.67 | 0.61 | 0.64 | 0.55 | 0.56 | **0.66±0.05** | **0.65±0.06** | **0.61±0.05** |
| LH_PrintArea_(cm²)_Mean | 0.27 | 0.27 | 0.25 | 0.36 | 0.28 | 0.29 | 0.30 | 0.31 | 0.29 | 0.24 | 0.25 | 0.28 | 0.24 | 0.30 | 0.27 | 0.22 | 0.20 | 0.25 | **0.29±0.04** | **0.28±0.03** | **0.25±0.04** |
| LH_MaxIntensityAt_(%)_Mean | 39.44 | 43.40 | 38.63 | 46.83 | 52.04 | 43.89 | 45.73 | 56.56 | 48.54 | 50.79 | 36.92 | 29.57 | 45.60 | 40.43 | 52.06 | 22.51 | 46.44 | 47.27 | **44.04±4.95** | **44.68±9.83** | **42.39±10.42** |
| LH_MaxIntensity_Mean | 196.00 | 191.90 | 184.69 | 201.77 | 181.36 | 185.46 | 196.68 | 203.50 | 176.02 | 182.99 | 194.08 | 196.90 | 153.13 | 160.03 | 183.09 | 179.02 | 171.48 | 166.69 | **190.19±7.76** | **191.69±10.18** | **168.91±11.34** |
| LH_MinIntensity_Mean | 36.35 | 36.67 | 36.80 | 36.04 | 36.49 | 36.93 | 35.97 | 36.26 | 35.88 | 36.74 | 36.15 | 35.67 | 36.84 | 36.71 | 36.04 | 37.13 | 36.42 | 36.28 | **36.55±0.32** | **36.11±0.37** | **36.57±0.4** |
| LH_MeanIntensity_Mean | 84.35 | 87.32 | 82.51 | 91.08 | 86.34 | 84.40 | 84.35 | 89.81 | 82.39 | 81.63 | 87.28 | 89.80 | 77.91 | 79.05 | 83.10 | 79.44 | 80.35 | 80.27 | **86±3** | **85.88±3.61** | **80.02±1.76** |
| LH_MeanIntensityOfThe15MostIntensePixels_Mean | 130.42 | 138.16 | 124.17 | 156.32 | 132.61 | 131.59 | 133.99 | 146.38 | 126.84 | 122.59 | 132.05 | 142.84 | 110.31 | 115.62 | 129.06 | 114.81 | 112.37 | 117.80 | **135.54±11.12** | **134.12±9.13** | **116.66±6.61** |
| LH_Swing_(s)_Mean | 0.17 | 0.14 | 0.14 | 0.10 | 0.34 | 0.09 | 0.14 | 0.11 | 0.30 | 0.15 | 0.19 | 0.27 | 0.13 | 0.12 | 0.18 | 0.10 | 0.15 | 0.11 | **0.16±0.09** | **0.2±0.08** | **0.13±0.03** |
| LH_SwingSpeed_(cm/s)_Mean | 68.16 | 54.38 | 86.24 | 69.93 | 45.41 | 99.90 | 61.17 | 63.87 | 55.26 | 69.19 | 59.29 | 49.84 | 67.49 | 94.35 | 43.26 | 89.15 | 51.90 | 61.49 | **70.67±20.04** | **59.77±6.73** | **67.94±20.27** |
| LH_StrideLength_(cm)_Mean | 9.98 | 7.27 | 10.18 | 6.71 | 12.48 | 8.54 | 7.89 | 6.74 | 12.33 | 10.05 | 10.51 | 12.98 | 8.30 | 8.88 | 6.38 | 7.03 | 7.14 | 6.78 | **9.19±2.13** | **10.08±2.43** | **7.42±0.96** |
| BOS_HindPaws_Mean_(cm) | 2.47 | 2.46 | 2.54 | 2.36 | 2.37 | 2.64 | 2.45 | 2.58 | 2.62 | 2.53 | 2.38 | 2.30 | 2.11 | 2.28 | 2.17 | 1.99 | 1.92 | 2.25 | **2.47±0.11** | **2.48±0.12** | **2.12±0.14** |
| PhaseDispersions_LH->RH_Mean | 43.75 | 50.33 | 53.74 | 48.66 | 43.37 | 52.70 | 49.22 | 48.98 | 38.20 | 30.39 | 41.36 | 40.36 | 47.45 | 47.44 | 48.89 | 51.39 | 45.40 | 51.86 | **48.76±4.4** | **41.42±7.09** | **48.74±2.5** |
| Couplings_LF->RH_CStat_R | 0.76 | 0.64 | 0.83 | 0.81 | 0.83 | 0.86 | 0.82 | 0.83 | 0.81 | 0.98 | 0.92 | 0.91 | 0.85 | 0.78 | 0.89 | 0.92 | 0.86 | 0.80 | **0.79±0.08** | **0.88±0.07** | **0.85±0.05** |
| Couplings_LH->RF_Mean | 72.03 | 56.12 | 61.55 | 62.38 | 68.99 | 54.86 | 51.70 | 97.86 | 79.79 | 88.59 | 86.02 | 91.00 | 79.33 | 73.26 | 100.22 | 83.27 | 80.40 | 63.02 | **62.65±6.82** | **82.49±16.21** | **79.92±12.28** |
| Couplings_RF->LF_Mean | 46.61 | 48.56 | 47.93 | 50.77 | 49.06 | 45.75 | 50.15 | 50.21 | 49.45 | 48.36 | 56.86 | 50.79 | 48.17 | 50.05 | 50.55 | 46.25 | 53.37 | 49.23 | **48.11±1.79** | **50.97±3** | **49.6±2.4** |
| Couplings_RF->LF_CStat_Mean | 46.93 | 48.87 | 47.93 | 50.79 | 48.99 | 45.69 | 50.16 | 50.20 | 51.39 | 48.24 | 34.40 | 49.93 | 47.81 | 50.82 | 50.53 | 45.46 | 53.37 | 49.05 | **48.2±1.77** | **47.39±6.44** | **49.51±2.73** |
| Couplings_RF->LF_CStat_R | 0.92 | 0.90 | 0.99 | 0.84 | 0.94 | 0.92 | 0.97 | 0.82 | 0.81 | 0.88 | 0.64 | 0.84 | 0.66 | 0.81 | 0.97 | 0.89 | 0.94 | 0.79 | **0.92±0.05** | **0.83±0.11** | **0.84±0.11** |
| Couplings_LH->LF_Mean | 49.13 | 54.13 | 55.78 | 51.18 | 49.10 | 52.07 | 57.75 | 50.84 | 65.17 | 47.33 | 57.98 | 67.60 | 44.96 | 53.55 | 45.07 | 50.08 | 51.09 | 52.32 | **51.9±2.69** | **57.78±7.85** | **49.51±3.67** |

1. Supplementary Figure S1：Design blueprint for the mouse survival box


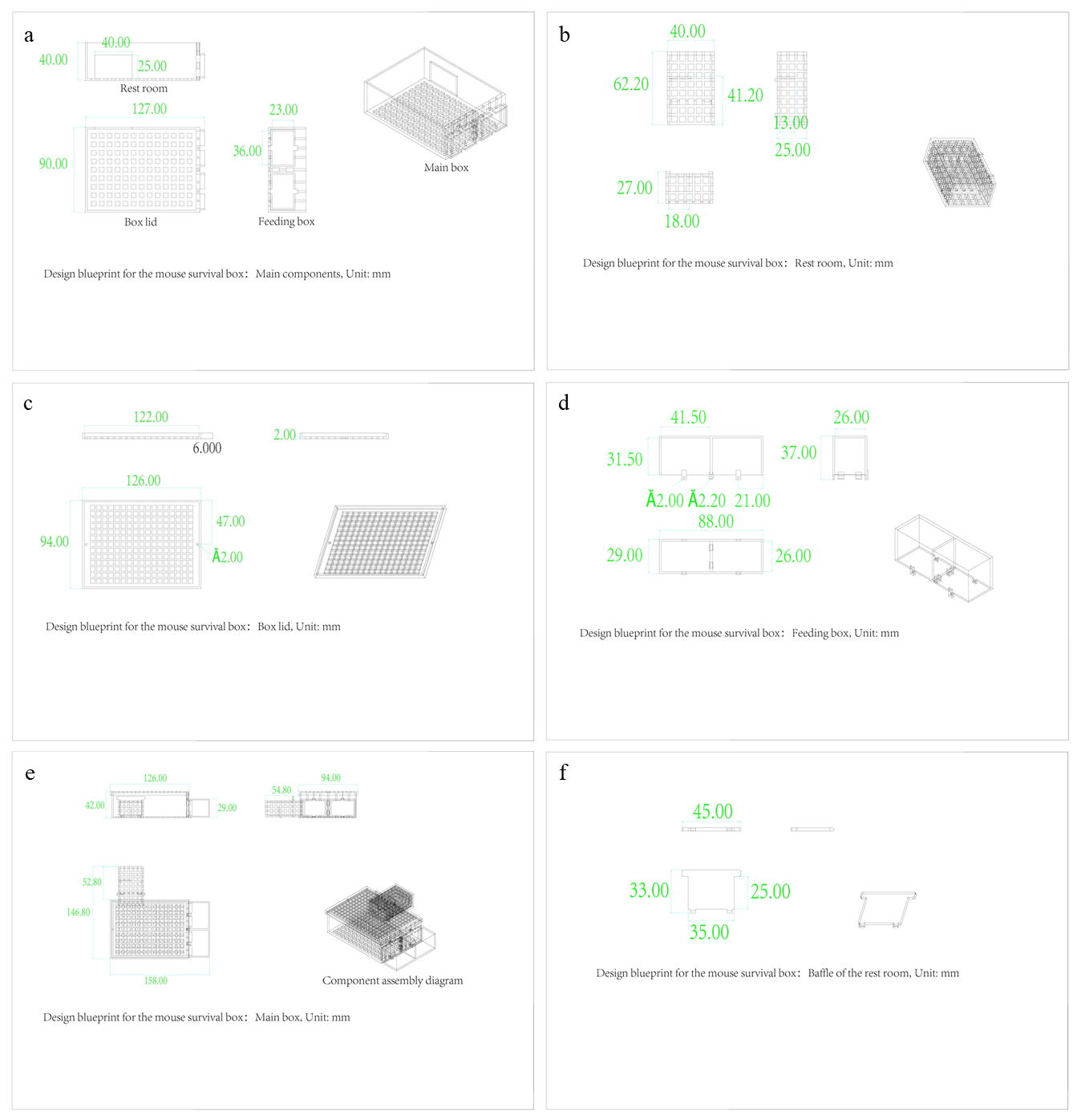


**Figure S1: Design blueprint for the mouse survival box.** (a) Main components; (b) Rest room; (c) Box lid; (d) Feeding box; (e) Main box; (f) Baffle of the rest room. Unit: mm
